# Time course of cisplatin-induced nephrotoxicity and hepatotoxicity

**DOI:** 10.15171/jnp.2017.28

**Published:** 2017-01-05

**Authors:** Zahra Pezeshki, Atoosa Khosravi, Mina Nekuei, Samaneh Khoshnood, Elnaz Zandi, Marjan Eslamian, Ardeshir Talebi, Seyyed Nasir-e-din Emami, Mehdi Nematbakhsh

**Affiliations:** ^1^Water & Electrolytes Research Center, Isfahan University of Medical Sciences, Isfahan, Iran; ^2^Department of Clinical Pathology, School of Medicine, Isfahan University of Medical Sciences, Isfahan, Iran; ^3^Department of Physiology, School of Medicine, Islamic Azad University, Najaf-Abad Branch, Najaf-Abad, Iran; ^4^Department of Physiology, School of Medicine, Isfahan University of Medical Sciences, Isfahan, Iran; ^5^IsfahanMN Institute of Basic & Applied Sciences Research, Isfahan, Iran Original Article

**Keywords:** Cisplatin, Nephrotoxicity, Hepatotoxicity

## Abstract

**Background:**

One of the main therapeutic limitations of cisplatin (CP) is nephrotoxicity which is time-dependent.

**Objectives:**

The purpose of this study was to determine the optimal timing for initiation of CP toxicity.

**Materials and Methods:**

Sixty male and female Wistar rats were randomly divided into five groups. All the animals in groups 2-5 received single dose of CP (10 mg/kg; i.p.), and were evaluated 25, 50, 75, and 100 hours after CP administration. Group 1 as an untreated group did not receive any agent and was considered as time zero.

**Results:**

The data indicated time-dependent progression of kidney and hepatic toxicity due to CP administration. Histological examination showed increase in kidney tissue damage score (KTDS) at hour 25, which peaked 75-100 hours after CP administration. Significant body weight loss and reduction of alkaline phosphatase (ALP) 50 hours after CP injection were observed. Blood urea nitrogen (BUN), creatinine (Cr), and serum nitrite increased significantly 75 hours after CP injection. Also, enhancement of kidney and testis weights, and alkaline aspartate aminotransferase (AST) level; and reduction of alanine aminotransferase (ALT) level and uterus weight occurred significantly 100 hours after the injection, while kidney malondialdehyde level enhanced significantly 75 hours after CP administration.

**Conclusions:**

These findings suggest that the CP-induced nephrotoxicity started to develop almost 3 days after administration of the drug in rats. CP surprisingly reduced the serum levels ALP and ALT while AST increased 100 hours after CP injection. CP-induced nephrotoxicity and hepatotoxicity are time-dependent, and the related biomarkers may alter by different trends.

**Implication for health policy/practice/research/medical education:**

In an experimental investigation on sixty male and female Wistar rats, we found CP induced nephrotoxicity started to develop almost three days after administration of the drug in rats. CP surprisingly reduced the serum levels ALP and ALT, while AST increased 100 hours after CP injection. CP induced nephrotoxicity and hepatotoxicity are time-dependent, and the related biomarkers may alter by different trends.

## 1. Background

Cisplatin (CP), an inorganic platinum chemotherapeutic drug, has been commonly used in a wide variety of solid tumors such as head, neck, lung, and breast cancers (1). Although CP has a suppressing effect on tumors, its use is limited due to severe side effects such as nephrotoxicity and hepatotoxicity. The major side effect of CP is nephrotoxicity (2). CP destroys cell membrane and induces tubular dysfunction through mechanisms such as production of reactive oxygen species (ROS) and hydroxyl radicals and inducing lipid peroxidation, inflammation, and hypoxia (1,3,4). All these injuries reduce glomerular filtration rate and induce acute nephrotoxicity (1). CP has been shown to accumulate into kidney to a greater degree than other organs (1). It has an initial plasma half-life of 25-49 minutes and a secondary half-life of 58-73 hours (5). CP side effects and the inhibitory effect of CP on tumor growth are time- and dose-dependent. It has been shown that administration of CP in low doses leads to apoptosis and high doses of CP cause necrosis (6,7). Moreover, optimal timing of drug administration is suggested for drugs with a narrow therapeutic range such as anticancer agents (8,9). Several studies have reported that CP nephrotoxicity is time-dependent, and by taking the effect of dose and time on CP-induced nephrotoxicity into consideration, CP administration can be performed with higher efficiency on tumor inhibition and less side effects (8,10,11).

## 2. Objectives

It is necessary to understand the optimal timing for development of CP side effects. So, this study was designed to identify the particular time for initiation of kidney or hepatic damage by CP.

## 3. Materials and Methods

Sixty adult male and female Wistar rats (178.55 ± 2.21 g) were individually housed at the temperature range of 23-26˚C and standard light cycle. The rats were randomly divided into five groups. Group 1 as an untreated group did not receive any agent (the biomarkers in these animals were considered as normal biomarkers at time zero). Rats in groups 2-5 received a single dose of CP (10 mg/kg; i.p.) at the beginning of the study and were sacrificed 25, 50, 75, and 100 hours after administration of CP, respectively. The number of animals analyzed in each group was 12 in groups 1, 2, and 3; 10 in group 4, and 14 in group 5. Finally, after taken of blood samples, the animals were sacrificed and kidneys, testis, and ovaries were removed and weighed immediately. The serum level of blood urea nitrogen (BUN), creatinine (Cr), alkaline phosphatase (ALP), alanine aminotransferase (ALT), and aspartate aminotransferase (AST) were measured by quantitative diagnostic kits (Pars Azmoon, Tehran, Iran). An assay kit was used for measuring serum and kidney levels of nitrite. Serum and kidney malondialdehyde (MDA) were assessed by manual method.

### 3.1. Ethical issues

The research followed the tenets of the Declaration of Helsinki. The research was approved by ethical committee of Isfahan University of Medical Sciences. Prior to the study, the protocol were confirmed to be in accordance with the Guidelines of Animal Ethics Committee of Isfahan University of Medical Sciences.

### 3.2. Statistical analysis

The data were analyzed by one-way analysis of variance (ANOVA) followed by the Dunnett test and regression analysis. Also, the Kruskal-Wallis and Mann-Whitney tests were used to compare the groups in terms of the pathology score. *P* values less than 0.05 were considered statistically significant.

## 4. Results

**Figure 1:**
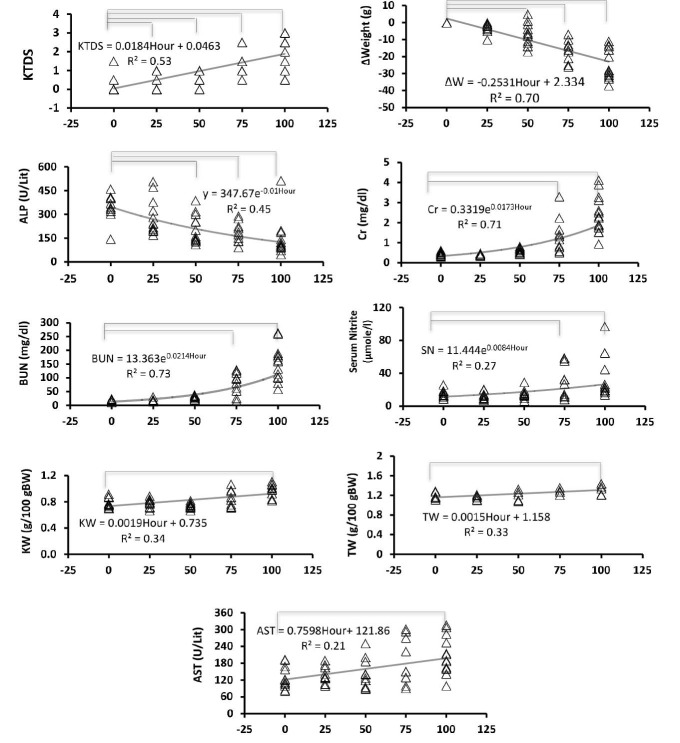
The trend of changes in the serum levels of BUN, Cr, nitrite, AST, ALP, KW, BW, TW, and KTDS in rats treated with single dose of CP at 25, 50, 75, and 100 hours after CP injection. Zero in horizontal axis indicates the data from normal animals. 25, 50, 75, and 100 in horizontal axis indicate the data from animals at 25,50, 75 and 100 hours after CP administration. The lines above each chart indicate significant difference from time zero (*P* < 0.05).

Abbreviations: TW, testis weight; BW, body weight; KW, kidney weight; KTDS, kidney tissue damage score; ALP, alkaline phosphatase; BUN; blood urea nitrogen; Cr, creatinine; CP, cisplatin.

**Figure 2:**
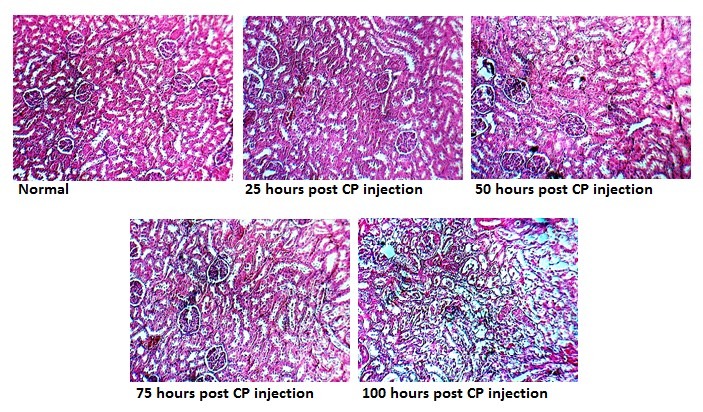
The images of kidney tissue stained with H&E to examine the kidney tissue damage.

The data indicated that CP-induced kidney and hepatic toxicity progressed in a time-dependent manner (Figure 1, Table 1). Most parameters started to change linearly or exponentially from day 2-3. Kidney level of nitrite and serum level of MDA did not alter significantly while some parameters such as AST and ALT changed 100 hours after CP injection . The sample images of kidney tissue are demonstrated in Figure 2.

**Table 1:** The serum levels of MDA and ALT and kidney levels of MDA and nitrite and uterus weight 25, 50, 75, and 100 hours after CP injection compared to time zero.

**Hour**	**Kidney nitrite (µmol/g tissue)**	**Serum MDA (µmol/L)**	**Kidney MDA (nmol/g tissue)**	**ALT (U/L)**	**Uterus weight (g/100 g BW)**
0	0.17 ± 0.01	6.25 ± 0.42	9.95 ± 0.74	54.92 ± 3.2	0.0614 ± 0.008
25	0.18 ± 0.01	5.69 ± 0.39	11.94 ± 0.45	47.25 ± 2.03	0.0480 ± 0.006
50	0.19 ± 0.01	6.45 ± 0.38	11.39 ± 0.55	39.92 ± 2.9	0.0531 ± 0.005
75	0.14 ± 0.01	5.90 ± 0.33	15.32 ± 1.73*	46.90 ± 8.5	0.0506 ± 0.004
100	0.18 ± 0.02	6.43 ± 0.40	10.53 ± 0.52	34.36 ± 6.9*	0.0372 ± 0.006*
*P*	0.105	0.560	0.001	0.064	0.102

Abbreviations: BW, body weight; ALT, alanine aminotransferase; MDA, malondialdehyde. * The star symbol indicates significant difference from time zero (*P* < 0.05).

## 5. Discussion

CP-induced nephrotoxicity is the major limitation for use of CP in tumor chemotherapy. Also, hepatic toxicity after CP injection is common (12). It is very important to monitor the CP side effects in patients under chemotherapy. The results of this study indicated time-dependent progression in kidney and hepatic toxicity due to CP administration. CP-induced kidney and hepatic toxicity were confirmed by time-dependent increase in BUN, Cr, KW, AST, serum nitrite, and kidney levels of MDA; and reduction in BW (12-16). In this study, BUN and Cr as the major biomarkers in kidney injury increased between hours 50 and 75 after CP injection while the kidney injury started 25 hours and peaked 75 hours after CP injection. According to the studies, changes in BUN and Cr levels (as conventional renal injury markers) occur with a delay after development of kidney injury (17,18). BUN and Cr levels show less sensitivity at the onset of kidney injury. This leads to delayed diagnosis and lead to miss early therapeutic interventions (19,20). Also, serum nitrite increases significantly 50-75 hours after CP injection as mentioned before (21). Body weight loss was seen at the beginning of the study and increased significantly 50 hours after CP injection. Reduce in body weight obeys a linear graph. It seems that body weight loss occurs by gastrointestinal disturbance (22) and enhancement of urine output after CP injection. It seems that body weight loss have a good concordance with the onset of CP side effects (18). In this study, KTDS altered earlier than other markers that we measured. It is reported that renal cellular disturbance is detected in early stage of CP damage (17,18). Direct examination of tissue pathology is very important to determine the side effect of CP on kidney in animal models. However, this cannot be routinely applied in the clinic. Accordingly, some investigations are necessary to monitor their clinical utility. A study have shown a seven-fold enhancement in urinary excretion of organic anion transporter type 5 two days after CP administration (5 mg/kg), while no variations were seen in conventional renal injury markers (18). Also, changes in urinary levels of Kim-1, α-GST, and albumin demonstrated to have good correlation with progression of CP-induced renal injury and their levels alter earlier than any conventional marker of renal injury (17). Other studies have shown a direct relation between kidney weight gain and increase in renal injury (23). However, we observed a delay in alteration of these indices in comparison with other markers. In patients with CP chemotherapy, hepatic toxicity appears after nephrotoxicity (2). Hepatotoxicity is determined by increase in serum AST level (12). The result of the present study indicated enhancement of AST 75-100 hours after CP injection. However, we found reduction of ALP and ALT levels 50 and 75 hours after CP administration, respectively. Administration of CP induces magnesium deficiency (24). A study indicated that serum ALP activity reduces due to magnesium depletion (25). At this time, we can state that reduction in levels of some liver enzymes by CP may be related to liver tissue damage. Nevertheless, further study is needed to determine the mechanism.

## 6. Conclusions

CP-induced nephrotoxicity and hepatotoxicity are time-dependent, and the related biomarkers may alter by different trends.

## Authors’ contribution

ZP designed and conducted the research and prepared the first draft of the article. AK, MN, SK, EZ and ME conducted the research, AT supervised and analyzed the pathology data. SNE designed and supervised the study. MN designed and supervised the study, analyzed the data and prepared the final draft of the article

## Conflicts of interest

The authors declared no competing interests.

## Funding/Support

This research was supported from Isfahan University of Medical Sciences (Grant #293362).
